# Multi‐Scale MXene/Silver Nanowire Composite Foams with Double Conductive Networks for Multifunctional Integration

**DOI:** 10.1002/advs.202403551

**Published:** 2024-06-13

**Authors:** Chenhui Xu, Zhihui Li, Tianyi Hang, Yiming Chen, Tianlong He, Xiping Li, Jiajia Zheng, Zhiyi Wu

**Affiliations:** ^1^ Key Laboratory of Urban Rail Transit Intelligent Operation and Maintenance Technology & Equipment of Zhejiang Province College of Engineering Zhejiang Normal University Jinhua 321004 China; ^2^ Beijing Institute of Nanoenergy and Nanosystems Chinese Academy of Sciences Beijing 100083 China

**Keywords:** composite foam, functional integration, MXene, silver nanowire

## Abstract

With the onset of the 5G era, wearable flexible electronic devices have developed rapidly and gradually entered the daily life of people. However, the vast majority of research focuses on the integration of functions and performance improvement, while ignoring electromagnetic hazards caused by devices. Herein, the 3D double conductive networks are constructed through a repetitive vacuum‐assisted dip‐coating technique to decorate the 2D MXene and 1D silver nanowires on the melamine foam. Benefiting from the unique porous structure and multi‐scale interconnected frame, the resultant composite foam exhibited high electrical conductivity, low density, superb electromagnetic interference shielding (48.32 dB), and Joule heating performance (up to 90.8 °C under 0.8 V). Furthermore, a single‐electrode triboelectric nanogenerator (TENG) with powerful energy harvesting capability is assembled by combining the composite foam with an ultra‐thin Ecoflex film and a polyvinylidene fluoride film. Simultaneously, the foam‐based TENG can also be considered a reliable wearable sensor for monitoring activity patterns in different parts of the human body. The versatility and scalable manufacturing of high‐performance composite foams will provide new design ideas for the development of next‐generation flexible wearable devices.

## Graphical abstract

An ultra‐flexible melamine composite foam decorated with conductive AgNWs and MXene is successfully fabricated by a facile dip‐coating strategy, which integrates the multifunction of ideal EMI shielding, impressive electro‐thermal conversion, and powerful energy harvesting capability in one, owing to the unique multi‐dimensional porous structure and effective conductive network inside the composite foam.

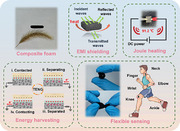

## 1 Introduction

The depletion of nonrenewable energy resources underscores the urgent need for sustainable and clean energy to achieve a balance between industrial progress and ecological preservation.^[^ ^1^ ^]^ As an innovative solution for harvesting green energy, triboelectric nanogenerator (TENG) offers numerous advantages, such as independence from external power sources, portability, and embeddability.^[^ ^2^, ^3^, ^4^ ^]^ By converting the frictional energy generated in daily activities into electrical power, TENG can provide sustainable support for most miniature electronic devices.^[^ ^5^ ^]^

Extensive research has focused on enhancing the output performances of TENGs for practical applications. Previous studies identified the material selection (e.g., polyethylene, polytetrafluoroethylene, polytetrafluoroethylene (PTFE), graphene, and metal oxides) and contact area as critical factors that influence the performances of TENG.^[^ ^6^, ^7^ ^]^ However, the choice of electrode layers for TENGs is very limited. Metal films like gold,^[^ ^8^ ^]^ aluminum,^[^ ^9^ ^]^ and copper^[^ ^10^ ^]^ are often chosen on account of their good conductivity. However, these metal layers are difficult to utilize in practical application scenarios due to their high density, poor flexibility, and difficult processing. On the contrary, polymer foams with low density, excellent mechanical properties, and flexibility have gained enormous attention.^[^ ^11^ ^]^ They can serve as a mechanical support layer due to their outstanding elasticity and ease of processing and modification. Weldemhret et al.^[^ ^12^ ^]^ coated the polyurethane foam with phosphorus‐doped mesoporous carbon as a TENG pole to gain excellent output performance. Chen et al.^[^ ^13^ ^]^ developed a wheat flour/carbon nanotube composite foam for energy harvesting and other functions. These demonstrate the great potential of foam‐based materials as TENGs to harvest green energy.

However, the heat generated by this high‐output energy harvesting TENG builds up in electronic components, severely reducing their reliability and lifetime.^[^ ^14^ ^]^ Additionally, the inevitable generation of electromagnetic radiation in electronic devices poses a significant risk of irreversible damage to human health following long‐term exposure. The excessive pursuit of performance improvement leads to neglecting the electromagnetic hazards caused by devices. The advent of the 5G era undoubtedly accelerates the vigorous development of the intelligence industry, and the ensuing electromagnetic pollution puts forward higher requirements for electromagnetic interference (EMI) shielding. Therefore, how to balance between the high performance of the devices and electromagnetic protection and multifunctional integration in the fields of energy harvesting,^[^ ^15^ ^]^ electromagnetic protection,^[^ ^16^ ^]^ flexible sensing,^[^ ^17^ ^]^ electronic skin,^[^ ^18^ ^]^ and artificial intelligence^[^ ^19^ ^]^ has become a more demanding issue. Creating an interface for impedance matching relies on the suitable electrical conductivity of materials to achieve EMI shielding and multifunctional integration, which is a potential approach to effectively address the above issue. Wang et al.^[^ ^20^ ^]^ developed a flexible multifunctional electronic device incorporating EMI shielding Joule heating, and strain sensing via coating technique and thermal crosslinking method. Cheng et al.^[^ ^21^ ^]^ engineered a 2D transition metal carbide/nitride (MXene)/carboxymethyl cellulose aerogel‐based TENG through freeze‐drying. The construction of 3D highly conductive networks could achieve effective coupling of EMI shielding, energy harvesting, and self‐powered sensing.

**Figure 1:**
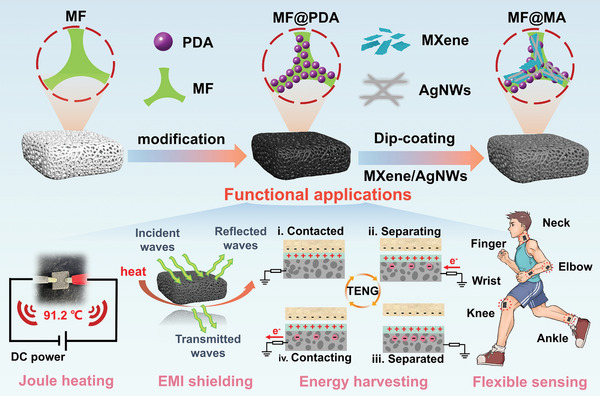
The schematic of the preparation process and functional applications of MF@MA.

In this work, a commercial melamine foam (MF) was successfully decorated with 2D MXene and 1D silver nanowires (AgNWs) using a simple vacuum‐assisted dip‐coating strategy (**Figure** **1**). The preparation process of the composite foams was simple and green, and the production cycle was very short while ensuring reproducibility and scalability. Benefiting from the unique porous structure and multi‐scale interconnected frame, the resultant composite foam exhibited high electrical conductivity and low density, which integrated energy harvesting, EMI shielding, and thermal management functions in one. The multi‐scale composite foam with continuous double conductive networks achieved an excellent EMI shielding performance of 48.32 dB. Additionally, under a low voltage of 0.8 V, the surface of the composite foam maintained a steady‐state temperature of 90.8 °C, showcasing a remarkable Joule heating capability. Interestingly, it was found that the steady‐state temperature of the composite foam at 0.9 V for the larger size remained the same as that of the smaller size, which could be maintained at 106 °C. Significantly, the conductive composite foam deposited with an ultra‐thin Ecoflex film was successfully used as the negative electrode of a single‐electrode TENG. Both experimental and simulation results indicated its impressive energy harvesting capacity. This foam‐based TENG was also further explored to charge commercial capacitors, illuminate light‐emitting bulbs, and monitor the activities of the human body. This work will offer inspiration and technological support for developing the next generation of wearable electronic devices to meet the needs of multiple applications in complex environments.

## 2 Experimental Section

### 2.1 Materials

The commercial MF was purchased from Shanghai Beiyou Building Material Co., Ltd (China). LiF‐HCl etched MXene (Ti_3_C_2_T_x_)^[^ ^22^ ^]^ and AgNWs synthesized using a polyol method^[^ ^23^ ^]^ were obtained following the procedure outlined in the previous reports. Dopamine hydrochloride (98%, molecular weight: 189.64) and Trometamol (Tris, molecular weight: 121.14) were provided by Shanghai Aladdin Biochemical Technology Co., Ltd (China). Hydrochloric acid (HCl, 36–38%) and ethanol were obtained from Jinhua Southeast Chemical Instrument Co., Ltd (China). All chemicals were of analytical grade without other purification.

### 2.2 Preparation of MF@MA

MF@polydopamine (PDA) (MF@PDA) was first prepared using the method reported in the Supporting Information, similar to the previously reported work.^[^ ^24^ ^]^ Then, MF@PDA was vacuum‐assisted and immersed in the ethanol dispersions of MXene and AgNWs (10 mg mL^−1^) for 30 min. Finally, the fully impregnated product was completely dried at 60 °C for 3 h to obtain the lightweight MF@PDA/MXene/AgNW (MF@MA) (Figure S1a, Supporting Information). The dip‐coating process was repeated four times, and the resulting samples were denoted as MF@MA1, MF@MA2, MF@MA3, and MF@MA4, respectively, to investigate the effects of impregnation times on the microstructure and properties of MF@MA.

### 2.3 Construction of MF@MA‐TENG

A layer of Ecoflex layer was coated on the surface of MF@MA via the facial casting of premixed Ecoflex A and B (1/1), which was used as the negative electrode of the single‐electrode TENG. Some materials (e.g., PTFE, Nylon, Kapton, polyethylene terephthalate (PET), fluorinated ethylene propylene (FEP), Cu, and polyvinylidene fluoride (PVDF)) were selected as the other friction layers, resulting the MF@MA‐based TENG (MF@MA‐TENG).

### 2.4 Characterizations

The crystal structures were examined using X‐ray diffraction (XRD, D8 Advance, Bruker, Germany). The elemental compositions and chemical structures were characterized using X‐ray photoelectron spectroscopy (XPS, ESCALAB 250Xi, Thermo Fisher Scientific, USA) and Fourier‐transform infrared spectroscopy (FTIR, Nicolet Nexus 670, USA), respectively. The microscopic morphologies of MXene and AgNWs were examined using transmission electron microscopy (TEM, JEM‐2100F, JEOL Japan Electronics Co., Ltd., Japan). The pore structures of the foams were observed using a combination of scanning electron microscopy (SEM, Hitachi S‐4800, COXEM, Japan) and energy‐dispersive X‐ray spectroscopy (EDS, EX‐25, HORIBA, Japan). The mechanical properties were measured using a universal testing machine (UTM4204, Sansheng Technology Co., Ltd., China).

The electrical conductivity (*σ*) of MF@MA was calculated using the following formula:

(1) $$\sigma \;\left(\frac{S}{\mathrm{cm}}\right)=\frac{L}{R\times S}\;$$

where length (*L*) and cross‐sectional area (*S*) were measured using a steel ruler. *R* was the resistance on both sides of the sample, which was determined using a multimeter (DT9205A, Shenzhen Snake Electronics Co., Ltd., China).

A vector network analyzer (VNA, ZVB20, Rohde & Schwarz, Germany) was employed to estimate the electromagnetic parameters of MF@MA in the frequency range of 8.2–12.4 GHz. The scattering parameters were gathered, specifically *S_11_* and *S_21_*. The total EMI shielding effectiveness (*SE*_T_), reflection loss (*SE*_R_), and absorption loss (*SE*_A_) were calculated along with the transmittance coefficient (*T*) and reflectance coefficient (*R*) using the equations below:

(2) $$R\;={\left|S_{11}\right|}^{2}$$

(3) $$T\;={\left|S_{21}\right|}^{2}$$

(4) $$S\;E_{R}=\;-10\mathrm{lo}g_{10}\left(1-R\right)$$

(5) $$S\;E_{A}=\;-10\mathrm{lo}g_{10}\left(\frac{T}{1-R}\right)$$

(6) $$S\;E_{T}=\;SE_{R}+SE_{A}$$

The Joule heating capacity of MF@MA was quantified using an infrared camera (VarioCAM, Jenoptik, Germany) with a DC power supply (HSPY‐200‐05, Beijing Hansheng Puyuan Technology Co., Ltd., China).

The electrical output performances of MF@MA‐TENG, including open‐circuit voltage, short‐circuit current, transferred charge, and charge/discharge behavior, were accurately determined using a programmable electrometer (model 6514, Keithley, USA). A linear motor (S0124/500, LinMot, Germany) was used to energize MF@MA‐TENG.

### 2.5 Informed Consent Statement

The volunteer provided informed consent to participate in the experimentation involving the monitoring of human motions using MF@MA‐TENG.

## 3 Results and Discussion

### 3.1 Morphology and Chemical Structure

**Figure 2:**
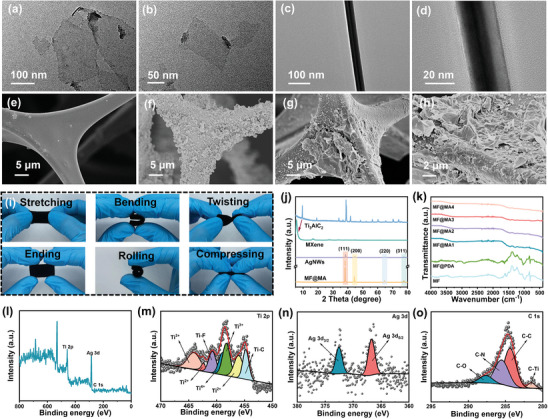
Morphologies and chemical structures of the composite foams. The TEM images of a,b) MXene and c,d) AgNWs. The SEM images of e) MF, f) MF@PDA, and g,h) MF@MA4. i) Stretchable, flexural, torsional, compressive, and crimp properties of MF@MA. j) XRD patterns and k) FTIR spectra of MF@MA. The XPS spectra of the MF@MA4: l) full spectrum, m) Ti 2p spectrum, n) Ag 3d spectrum, and o) C 1s spectrum.

The monolayer MXene with abundant surface functional groups was first prepared using the LiF‐HCl etching method.^[^ ^25^, ^26^, ^27^ ^]^ The clear sheet‐like outline of Ti_3_C_2_T_x_ with a large lateral dimension can be observed in **Figure** **2**a,b. AgNWs with a high aspect ratio were obtained using a simple polyol method, and the surface of AgNWs was coated with a thin layer of polyvinyl pyrrolidone (PVP) (Figure 2c,d). Compared to the smooth skeleton of the pure MF (Figure 2e), the MF surface became exceptionally rough after encapsulating by PDA (Figure 2f). Additionally, PDA was also a versatile adhesive that could self‐polymerize and be used for hydrophilic functional modification of almost all organic and inorganic materials. Contributing to the co‐existence of catechol (DOPA) and amine (lysine) groups, MF@PDA had enough adhesion ability to combine with functional nanofillers.^[^ ^28^ ^]^ The nanomaterials and MF were tightly bonded together due to these abundant surface functional groups of MXene, PVP, and PDA (Figure 2g,h). Moreover, with the increase in impregnation times, the number of conductive nanomaterials coated on MF significantly increased (Figure S1, Supporting Information). The elemental mappings confirmed the uniform distribution of Ag, Ti, C, O, and N elements (Figure S2, Supporting Information) indicating the successful assembly of the synthesized MXene and AgNWs on MF.

In the XRD patterns (Figure 2j), the vanishing of the diffraction peak of Ti_3_AlC_2_ at 2θ of 38.9° and a shift in the (002) diffraction peak from 9.7 to 5.6° suggested the removal of Al layers and the expansion of the layer spacing of MXene, indicating the successful preparation of MXene.^[^ ^29^ ^]^ Besides, the distinct peaks at 2θ of 38.1, 44.3, 64.5, and 77.5° of MF@MA correlated with the (111), (200), (220), and (311) planes of AgNW crystals (PDF#04‐0783), respectively. FTIR results showed that the peaks at 750, 1620, and 3430 cm^−1^ corresponded to C─F, Ti─O, and ─OH (Figure 2k), respectively, validating the presence of MXene in MF@MA.^[^ ^30^ ^]^ In addition, the XPS curves showcased the presence of Ti, Ag, and C elements within the composite foam (Figure 2l). The high‐resolution Ti 2p spectrum obtained from MF@MA showed distinct peaks corresponding to Ti─F, Ti─C, Ti^2+^, and Ti^3+^, consistent with prior research findings (Figure 2m).^[^ ^31^ ^]^ Two prominent peaks of Ag 3d at 367.2 and 373.2 eV were ascribed to Ag 3d_5/2_ and Ag 3d_3/2_, respectively, signifying the effective integration of AgNWs (Figure 2n).^[^ ^32^ ^]^ Additionally, the C 1s spectrum displayed four clear peaks at 289.1, 286.5, 285.1, and 281.8 eV, aligning with C─O, C─N, C─C, and C─Ti, respectively (Figure 2o).^[^ ^33^ ^]^

The excellent compression resistance of the MF skeleton and the good combination between the 3D skeleton and nanomaterials made the mechanical properties of the composite foam trustworthy (Figure 2i). After stretching and folding, the ending state of MF@MA was exactly the same as the initial state (Video S1, Supporting Information). The compressive stress of MF@MA at 80% strain improved with the increase of impregnation times (Figure S3a, Supporting Information), manifesting that the continuous dip‐coating of MXene and AgNWs had a positive effect on the mechanical properties of MF@MA. After ten compression‐release cycles, the cycling curves of the MF@MA4 basically overlapped except the first time at 40% strain (Figure S3b, Supporting Information). This indicated the stable compression recovery property of the composite foam.

### 3.2 EMI Shielding of MF@MA

**Figure 3:**
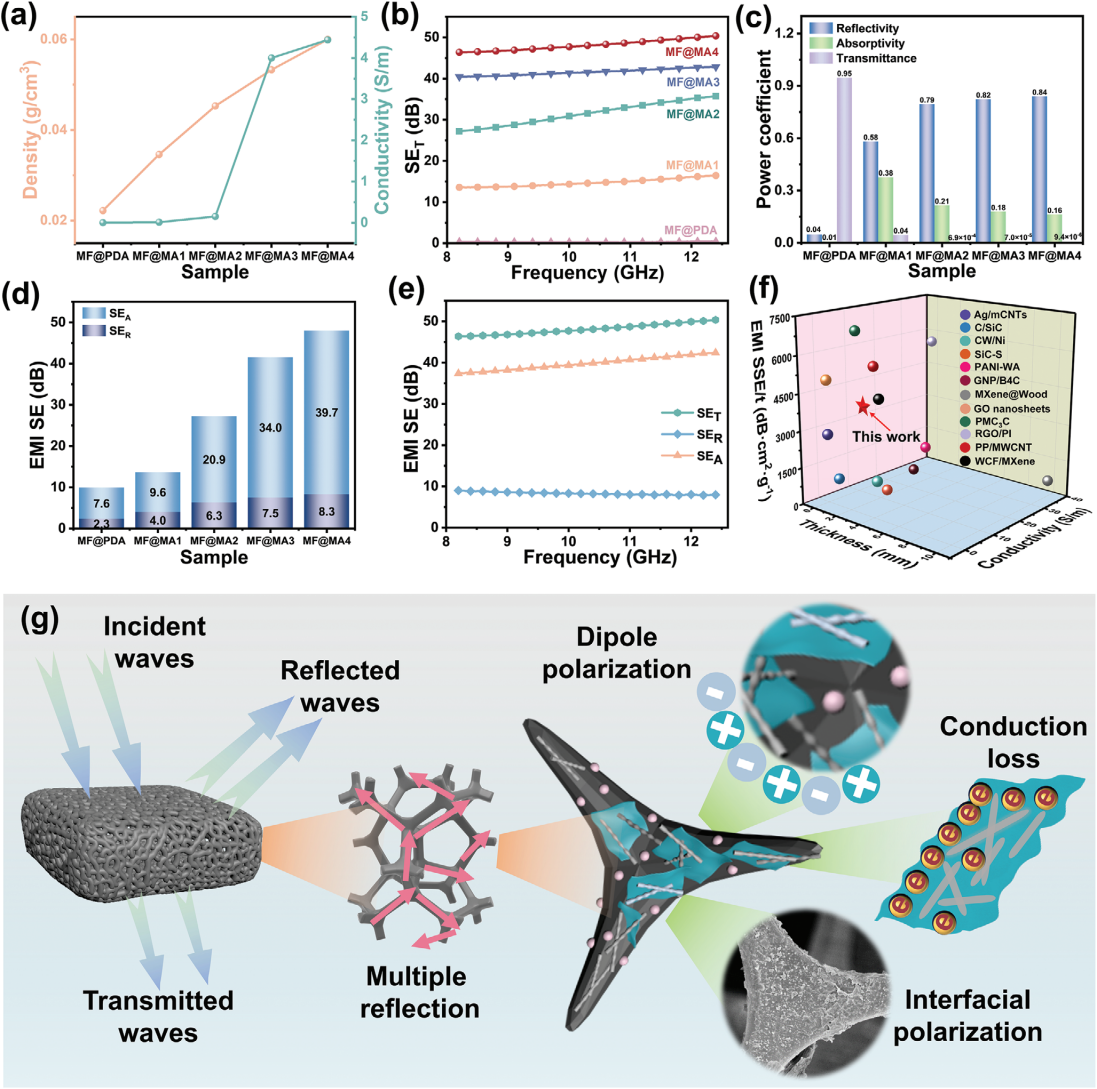
EMI shielding performance of MF@MA. a) The electrical conductivity and density, b) EMI SE_T_, c) power coefficients, and d) average SE_T_, SE_A_, and SE_R_ values of MF@MA. e) The SE_T_, SE_A_, and SE_R_ values of the MF@MA4. f) SSE/t versus thickness and conductivity of MF@MA compared with those of materials reported in the literature. g) The EMI shielding mechanisms of MF@MA.

Through repeatedly immersing MF in the MXene/AgNW mixed dispersion, MF@MA exhibited efficient EMI shielding performances due to the increased electrical conductivity of the composite foam.^[^ ^34^ ^]^ The electrical conductivity of MF@MA rose from 0.01 to 4.4 S/m with the progressive coating of highly conductive nanomaterials (**Figure** **3**a; Table S1, Supporting Information). The continuous accumulation of 1D and 2D conductive nanomaterials gradually constructed a complex multi‐dimensional interconnected conductive network inside the 3D MF skeleton, leading to the leap forward of the electrical conductivity of the composite foam. However, the contents of conductive fillers on the MF skeleton surface tended to be saturated from the MF@MA3 to MF@MA4, resulting in stagnation in the electrical conductivity increment. In addition, it could be easily observed that the SE_T_ values of MF@MA significantly rose with an increase in the immersion times. The average EMI SE_T_ of the MF@MA3 and MF@MA4 reached 41.72 and 48.32 dB (Figure 3b,e), respectively, which far exceeded the commercial standard (>20 dB) and was superior to other materials in the previous literature (Figure S4 and Table S2, Supporting Information). In order to meet the practical application, especially for the miniaturization of electronic devices, a high EMI SE value is not the only factor for EMI shielding materials; The density and thickness of the material should also be considered. The value of SSE/t is more appropriate to judge the EMI shielding performance. MF@MA3 and MF@MA4 showed an excellent EMI shielding performance and their values of SSE/t could reach 3935.84 and 4026.67 dB cm^−2^ g^−1^. Compared with other reported EMI shielding materials (Figure 3f; Table S3, Supporting Information), our values of SSE/t were not optimal but still competitive, and it was worth mentioning that our composite foam had many other advantages. For example, the preparation process of our material was simpler and greener than that of other EMI shielding materials, accompanied by more functional applications in complex environments. Benefiting from the unique porous structure and multi‐scale interconnected frame, the resultant composite foam exhibited high electrical conductivity and low density. Overall, our prepared composite foam demonstrated enormous potential as flexible and lightweight high‐performance EMI shielding materials in practical applications. Combining 1D AgNWs and 2D MXene could create numerous heterogeneous interface structures, greatly enhancing the SE_A_ values and strengthening the EMI shielding performance (Figure 3d).^[^ ^35^, ^36^ ^]^ In addition, the SE_R_ values showed the same changing trend as SE_A_, owing to the increased ohmic losses during the interaction with the incident electromagnetic waves.^[^ ^37^ ^]^

In addition, the effects of electromagnetic wave transmission, reflection, and absorption could be expressed by the power coefficients (Figure 3c).^[^ ^38^ ^]^ All MF@MA showed high R values, and the ratio between R and A gradually increased with more impregnations, revealing that the reflection loss played a dominant role in EMI shielding. Above all, the T value of the MF@MA4 was lower than 10^−5^. This meant that only a very small number of electromagnetic waves passed through the composite foam, reflecting its powerful EMI shielding capability.

Thereby, the EMI shielding mechanisms of MF@MA were explained in Figure 3g. First, when electromagnetic waves initially contact the MF@MA surface, substantial wave reflection will occur due to the high electrical conductivity of the composite foam. Subsequently, the residual electromagnetic wave enters the interior of MF@MA, where they are reflected and absorbed multiple times at internal interfaces of the composite foam owing to its porous and multi‐dimensional interconnected double network structure. Among other factors, interfacial polarization, dipole polarization, and conduction losses lead to the occurrence of polarization relaxation phenomenon,^[^ ^39^, ^40^ ^]^ which further promotes the dissipation of electromagnetic wave energy. Abundant movable charge carriers on the conductive network experienced shifts under the influence of electromagnetic waves, converting to internal energy dissipation, thus enhancing EMI shielding performance.^[^ ^41^ ^]^ The combination of several loss mechanisms facilitated effective reflection and absorption, helping the majority of electromagnetic waves isolate from MF@MA. Overall, the EMI shielding mechanism of MF@MA is a complex multifactorial process involving multiple aspects of electromagnetic wave reflection, absorption, interface polarization, and conduction loss. The interaction of these mechanisms together promotes the excellent performance of MF@MA in the field of electromagnetic protection.

### 3.3 Thermal Management of MF@MA

**Figure 4:**
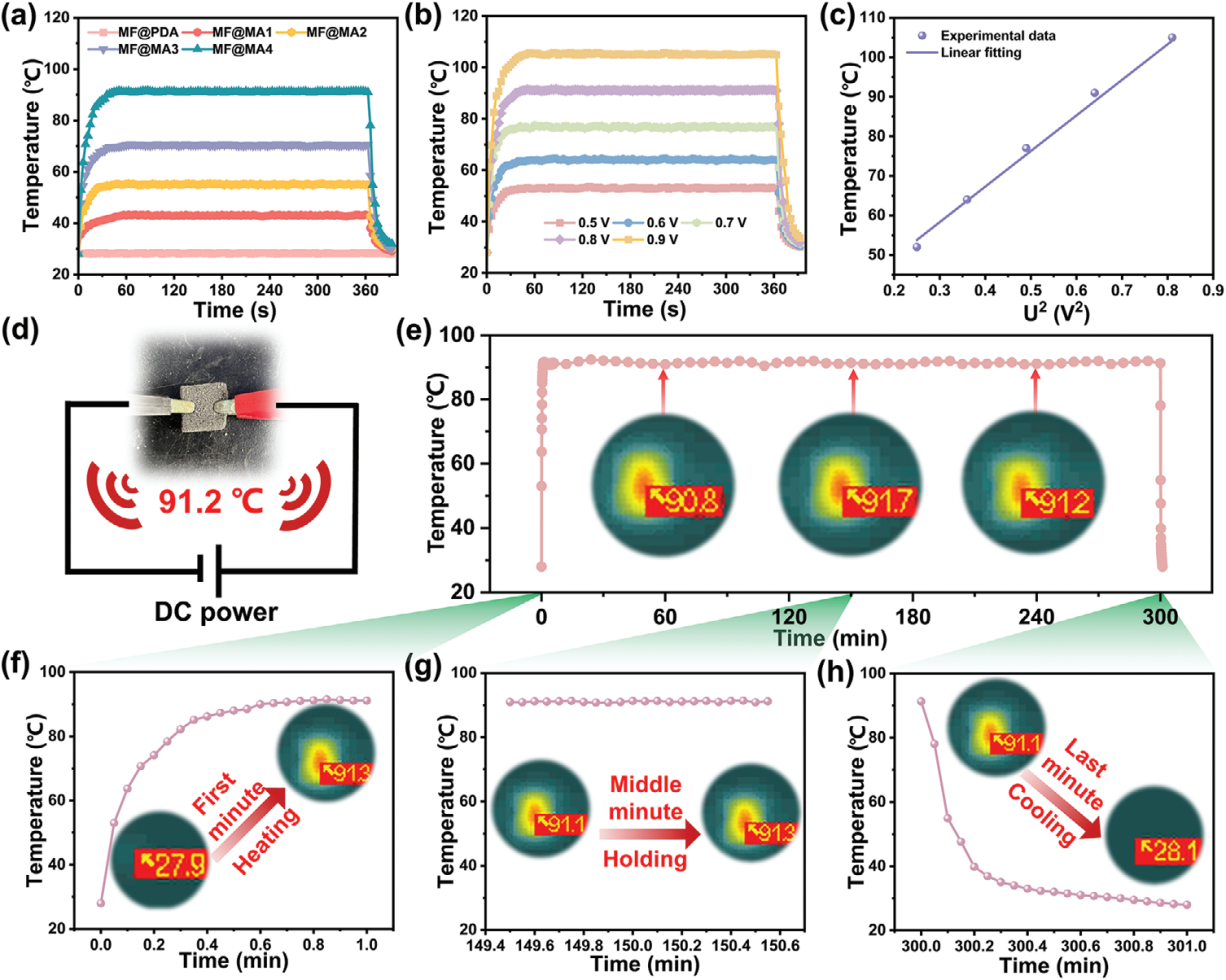
Thermal management of MF@MA. a) The time‐dependent surface temperatures of MF@MA under 0.8 V. b) The time‐dependent surface temperatures of the MF@MA4 at different voltages. c) The surface temperature changes of the MF@MA4 under stepwise voltage increase. d) The schematic diagram of the circuit. e) The 5‐h working stability testing of the MF@MA4 under 0.8 V at f) 1 min, g) 150 min, and h) 300 min.

The multi‐dimensional interconnected conductive network not only endowed the composite foam with strong EMI shielding properties but also made it suitable for personal thermal management. The temperature profiles of MF@MA under a low voltage of 0.8 V were captured by an infrared imaging camera (**Figure** **4**a). As expected, the electrically insulated MF@PDA showed no significant temperature change due to the absence of incorporated conductive fillers.^[^ ^42^ ^]^ The heating response speed of the composite foam increased progressively with the gradual completion of an effective conductive network. Notably, the MF@MA4 experienced a rapid temperature rise from room temperature to 85.1 °C within just 21 s. In addition, the temperature changes of the MF@MA4 under various low applied voltages are shown in Figure 4b. It was significant that a tiny increase in the applied voltage (0.1 V) led to a big leap forward in temperature, showcasing the high sensitivity of the Joule heating functionality. In comparison to similar studies,^[^ ^43^ ^]^ the MF@MA4 demonstrated the ability to reach higher temperatures under lower external voltages, emphasizing the significant advantage of the MF@MA4 over other materials. Consequently, it was simple to delineate the correlation between the applied voltage and the resultant steady‐state temperature (Figure 4c). The observed results indicated a nearly linear relationship between the steady‐state temperature and the square of the applied voltage. This phenomenon suggested that the applied voltage could directly impact the temperature, providing valuable insights for applications on controlled thermal management. The circuit diagram more visually showed that the MF@MA4 surface reached a steady state temperature with an external DC power supply (Figure 4d). Furthermore, maintaining the surface temperature of the MF@MA4 at ≈90.8 °C for 5 h, demonstrated its outstanding long‐term working stability (Figure 4e–h). Also, we re‐experimentally prepared an oversized MF@MA4 with a size of 100 mm × 100 mm (Figure S5, Supporting Information). Interestingly, it was found that the steady‐state temperature of the MF@MA4 at 0.9 V for the larger size remained the same as that of the smaller size, which could be maintained at 106 °C (Video S2, Supporting Information). It could be shown that MF@MA4 at a larger size still had good Joule heating performance, which further proved the excellence and uniqueness of our material.

Besides, the remarkable thermal insulation performance is a crucial feature for flexible wearable electronic devices to effectively protect human skin from overheating or excessive cold. The exceptional thermal stability of MXene, the unique 3D porous structure, and the low‐density air filling of the composite foam^[^ ^39^ ^]^ contributed to the significant potential of MF@MA in high‐temperature protection applications. When subjected to a heating platform with a temperature range of 75 to 200 °C, the temperature of the MF@MA4 increased from 50.3 to 114 °C (Figure S6, Supporting Information). These results indicated that the temperature difference between the two surfaces tended to be more pronounced with the increase in the temperature of the heating platform, highlighting the excellent thermal insulation properties of MF@MA.

### 3.4 Energy Harvesting and Sporting Monitoring of MF@MA‐TENG

**Figure 5:**
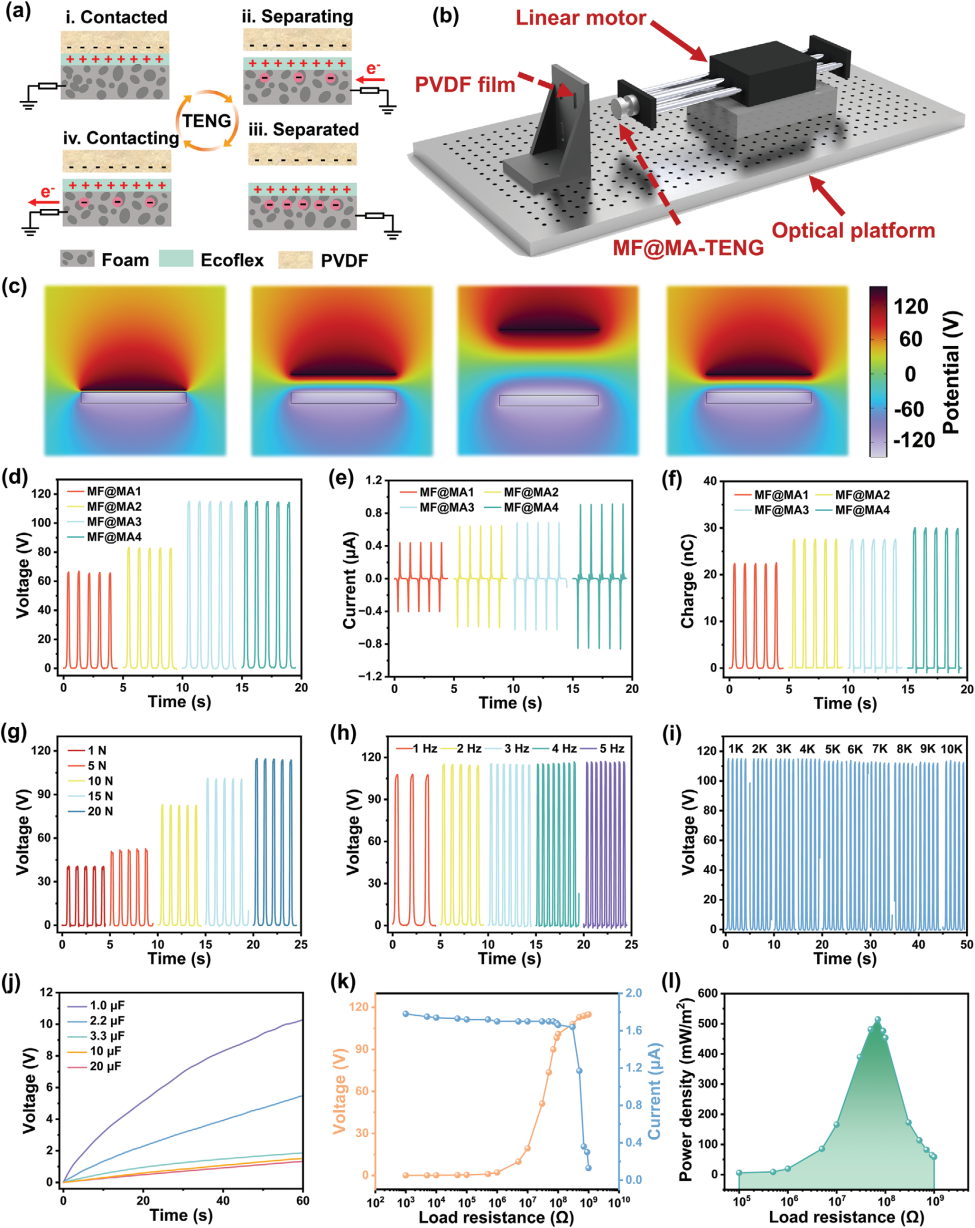
Energy harvesting of MF@MA‐TENG. a) The working mechanism and b) the operational schematic diagram of MF@MA‐TENG. c) Simulation‐derived potential distribution of the relevant stages by using COMSOL software. d) The open‐circuit voltage, e) the short‐circuit current, and f) the transferred charge amount of MF@MA‐TENG. The open‐circuit voltage of MF@MA4‐TENG at different g) contact pressures and h) frequencies. i) Working durability of MF@MA4‐TENG under a pressure of 20 N for 10 000 cycles. j) The charging curves of MF@MA‐TENG for different capacitors. k) The peak output voltage and current of MF@MA4‐TENG. l) The peak power density of MF@MA4‐TENG under various external resistances.

In addition to excellent EMI shielding and thermal management features, the real‐time monitoring of the human body is of great significance for wearable devices. Thanks to the excellent electrical conductivity and good mechanical properties, MF@MA could serve as a TENG electrode to provide ion transport for friction charges.^[^ ^44^ ^]^ In the single‐electrode TENG, the negative electrode was fabricated by applying an Ecoflex layer onto the MF@MA surface. When the positive material (PVDF film) made full contact with the negative material (Ecoflex layer) under external mechanical force, both materials underwent the generation of surface charges through the triboelectric effect (process (i) in **Figure** **5**a). The surfaces of Ecoflex and PVDF distributed both positive and negative friction charges equally. As the PVDF and Ecoflex separated, the partial neutralization of the positive charges on Ecoflex by the negative charges on PVDF caused the attraction of negative ions from MF@MA to the upper surface of Ecoflex (process (ii) in Figure 5a). Consequently, the electrons flowed from the external circuit to the foam. Once PVDF and Ecoflex were sufficiently separated, the positive charges on Ecoflex were fully shielded by the negative ions in MF@MA, leading to the cessation of charge movement in the circuit (process (iii) in Figure 5a). Finally, when PVDF approached Ecoflex, the potential difference between the two materials decreased. The improved electrostatic shielding deflected negative ions away from the surface of MF@MA, causing surplus electrons in the external circuit to discharge into the ground (process (iv) in Figure 5a). When the PVDF film made contact with Ecoflex again, the operating mode reverted to the initial state (i), thus perpetuating the cycle.

We conducted a series of tests for the output performance of MF@MA‐TENG by setting up a combination of linear motor and force sensor (Figure 5b). First, we selected seven common friction materials to assess their effects on the output performance of MF@MA‐TENG, including PTFE, Nylon, Kapton, PET, FEP, Cu, and PVDF (Figure S7, Supporting Information). Among these materials, the TENG with the PVDF film as the other friction layer achieved the largest output effect. This proved that the appropriate selection of friction materials could significantly enhance output performance. This was due to the extremely strong electron‐loss ability of the PVDF film, which matched the electron‐donating capability of Ecoflex.^[^ ^45^ ^]^ The potential changes during the contact separation process between the Ecoflex and the PVDF film were simulated using COMSOL software to further quantify the potential changes around MF@MA‐TENG (Figure 5c). Initially, when the two materials came into contact, most of the electrostatic charges accumulated on the surface of the friction layer, resulting in a minimal number of induced charges on the PVDF film. Consequently, the potential at this stage was relatively low. However, as the separation between the materials increased, there was an enhancement in the electrode charges due to the developing potential difference, leading to a peak voltage of ≈115 V. This value was consistent with the observations from the experimental setup, aligning well with the experimental results. With more impregnation times, the output voltages for MF@MA increased from 66.4 to 115.5 V (Figure 5d). Correspondingly, the output current and charge for the composite foams with different impregnation times exhibited a similar output trend (Figure 5e,f). This was because the gradually improved conductivity helped less electrical energy to be consumed in the electrode.

Additionally, the output performances of MF@MA‐TENG were investigated under different external mechanical forces and impact frequencies. The results showed that a greater external mechanical force could significantly improve the output effects of MF@MA‐TENG (Figure 5g). This improvement could be attributed to the increased proximity of contact between the poles as the external force intensified, thus enhancing electron exchange efficiency.^[^ ^46^ ^]^ As for the change of frequency, we found that it did not have a great impact on the TENG output; the open‐circuit voltage was maintained ≈115 V at different frequencies (Figure 5h). Moreover, when subjected to a 20 N applied force at a frequency of 2 Hz, the signal analysis of 10 000 cycles showed a relatively stable voltage, guaranteeing the long‐term effectiveness and reliability of MF@MA‐TENG (Figure 5i).

The charging rate was assessed on commercial capacitors of varying capacities to showcase the potential of MF@MA‐TENG in energizing devices (Figure 5j). Remarkably, MF@MA‐TENG was able to rapidly charge a 1 µF commercial capacitor from 0 to 12 V within 60 s. Furthermore, the maximum power output of MF@MA‐TENG was determined by measuring the voltage and current across a range of load resistances, ranging from 1 kΩ to 1 GΩ (Figure 5k). It was noted that as the load resistances changed, the current and voltage exhibited an opposite trend. The peak power density hindered by Ohmic losses reached its saturation point at ≈70 MΩ, registering at 514.2 mW m^−2^ (Figure 5l), which was satisfactory and showed significant advantages compared to other reported TENGs (Table S4, Supporting Information). In addition, the MF@MA‐TENG (60 mm × 60 mm × 2 mm) could be clearly seen to be able to light up all the LEDs indicated by the arrows at the same time (Figure S8 and Video S3, Supporting Information). Also, the MF@MA‐TENG (15 mm × 15 mm × 2 mm) could power a thermohygrometer (Figure S9 and Video S4, Supporting Information). Therefore, this MF@MA‐TENG demonstrates great potential in energy harvesting and powering microelectronic devices.

**Figure 6:**
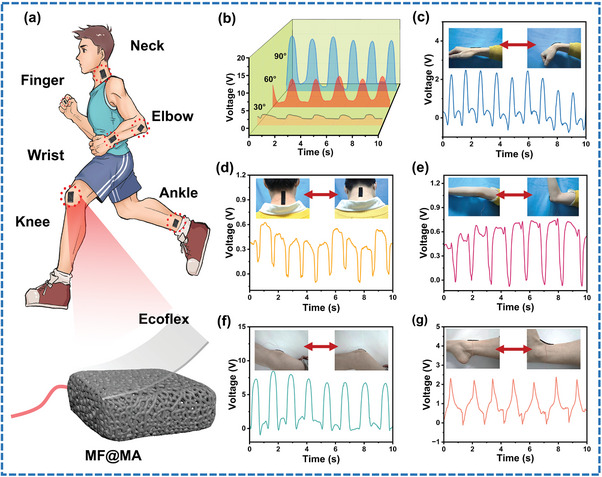
Human Sporting monitoring of MF@MA‐TENG. a) The structural diagram of the sensor and the schematic diagram of behavior monitoring. b) Voltage signals of the sensor yielded by finger bending at different angles. Voltage signals of the sensor in response to continuous activities of c) wrist, d) neck, e) elbow, f) knee, and g) ankle.

MF@MA‐TENG could be applied as a human motion monitoring sensor through continuous, real‐time, non‐invasive mode due to its excellent flexibility and efficient electrical output capability.^[^ ^47^ ^]^ The negative electrode of MF@MA‐TENG was attached to the human skin to generate corresponding electric signals when human body movements occurred. Therefore, the active modes of finger, wrist, neck, elbow, knee, and ankle could be monitored by the foam‐based wearable sensor (**Figure** **6**a). As the joint bent, the frictional electric pairs between the skin and the Ecoflex layer came into contact, generating friction charges at the interface. Further, the Ecoflex layer drove MF@MA to undergo compressive deformation while producing induced charges internally. As the joint stretched, the compressive deformation was released, and the accumulated charge was output through an external circuit.^[^ ^48^ ^]^

Figure 6b depicts the attachment of the sensor to the finger and its utilization for tracking finger motions. The flexion angles of the finger flexion angles were ≈30, 60, and 90°, resulting in output voltages of ≈2.5, 8, and 15 V, respectively. This change was attributed to the significant contact force applied between the sensor and the finger, demonstrating the precise ability of the sensor ability to detect bending angles of the finger. This research had potential applications in monitoring finger movements for tremor disorders such as Parkinson's syndrome. Figure 6c displays the output voltages of the sensor secured on the wrist. The voltage waveform exhibited good reproducibility for the same wrist flexion, indicating the ability of the sensor to detect subtle movements originating from muscles and tendons. Similarly, more substantial movements could be monitored by attaching the sensor to the elbow (Figure 6e) or knee (Figure 6f). By affixing the sensor to areas of the neck (Figure 6d) and ankle (Figure 6g) with limited mobility, the sensor was equally responsive to monitoring the movement of both. The sensor showed promising potential for detecting both extensive and nuanced movements and has significant applications in the field of robotic prosthetics and monitoring rehabilitative motions.^[^ ^49^ ^]^ The excellent EMI shielding and human motion sensing capabilities of the composite foam presented the possibility of simultaneous monitoring and safeguarding of the electromagnetic health of humans.^[^ ^21^, ^50^ ^]^

## 4 Conclusion

In summary, the flexible MF decorated with 2D MXene and 1D AgNWs was successfully prepared through a repetitive vacuum‐assisted dip‐coating method. The 3D continuous conductive networks derived by the multi‐dimensional MF@MA endowed the composite foam with competitive electrical conductivity. Owing to the characteristics of the double conductive networks and excellent mechanical properties, the multifunctional integration of the composite foam was realized: excellent EMI shielding efficiency (48.32 dB), outstanding electrothermal conversion functionality (90.8 °C under 0.8 V), extremely high electrical output (514.28 mW m^−2^) as a pole of TENG, and effective monitoring of human activities. Such an outstanding high‐performance composite foam establishes its strengths and possibilities in the field of wearable electronic devices and offers valuable insights for the development and implementation of multi‐scale advanced functional materials.

## Conflict of Interest

The authors declare no conflict of interest.

## Supporting information

Supporting Information

Supplemental Video 1

Supplemental Video 2

Supplemental Video 3

Supplemental Video 4

## Data Availability

The data that support the findings of this study are available from the corresponding author upon reasonable request.
