# Supplementary material for: Multi‐Scale MXene/Silver Nanowire Composite Foams with Double Conductive Networks for Multifunctional Integration
Source: Adv Sci (Weinh). 2024 Jun 13;11(30):2403551. doi: 10.1002/advs.202403551 (PMC11321636; doi:10.1002/advs.202403551)
Supplement: Supplementary file 1 — Supporting Information [file ADVS-11-2403551-s005.docx]

**Supporting information**

**Multi-scale MXene/silver nanowire composite foams with double conductive networks for multifunctional integration**

*Chenhui Xu^a,b^, Zhihui Li^a,b^, Tianyi Hang^a^, Yiming Chen^a,^*, Tianlong He^a^, Xiping Li^a^, Jiajia Zheng^a,^*, Zhiyi Wu^b,^**

Mr. C. Xu, Mr. Z. Li, Mr. T. Hang, Dr. Y. Chen, Mr. T. He, Prof. X. Li, Prof. J. Zheng

Key Laboratory of Urban Rail Transit Intelligent Operation and Maintenance Technology & Equipment of Zhejiang Province, College of Engineering, Zhejiang Normal University, Jinhua 321004, China.

E-mails: yiming.chen@zjnu.edu.cn (Y. Chen); jiajia.zheng@zjnu.cn (J. Zheng)

Prof. Z. Wu

Beijing Institute of Nanoenergy and Nanosystems, Chinese Academy of Sciences, Beijing 100083, China.

E-mail: wuzhiyi@binn.cas.cn (Z. Wu)

**Experiment section**

**Preparation of MF@PDA:** Initially, Tris (0.4 g) was dissolved in deionized water (200 mL), adjusting the pH to 8.5 with HCl (0.5 M). Following thorough mixing, dopamine hydrochloride (0.4 g) was introduced into the mixed solution. Meanwhile, MF was cut to a desired dimension of 15 mm×15 mm and was put into the above mixture. After stirring for 12 h, MF@polydopamine (PDA) was dried.

**Figures and tables**


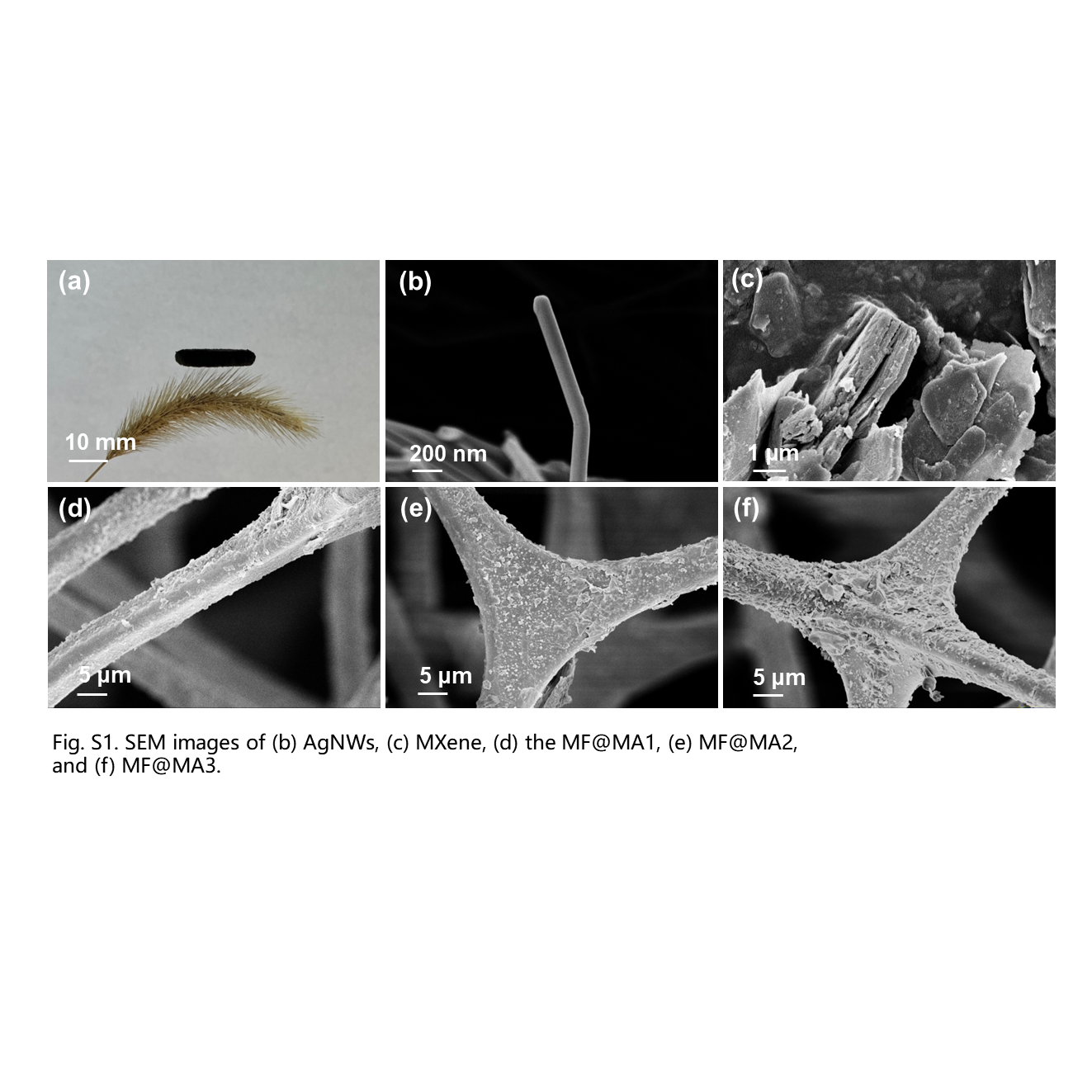


**Figure S1**. (a) Appearance of the ultra-light MF@MA. The SEM images of (b) AgNWs, (c) MXene, (d) the MF@MA1, (e) MF@MA2, and (f) MF@MA3.


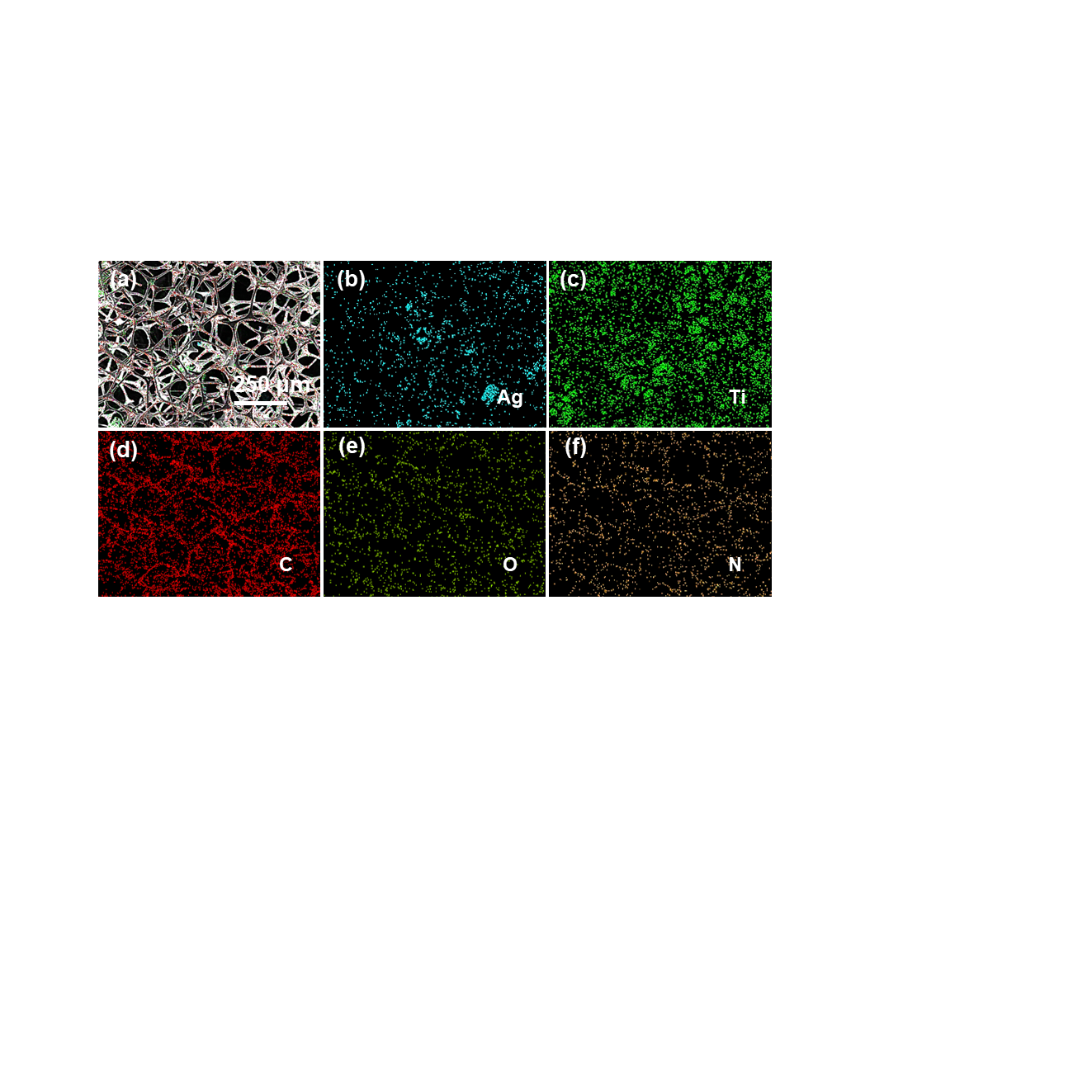


**Figure S2**. (a) The EDS mapping of MF@MA with element distribution images of (b) Ag, (c) Ti, (d) C, (e) O, and (f) N.


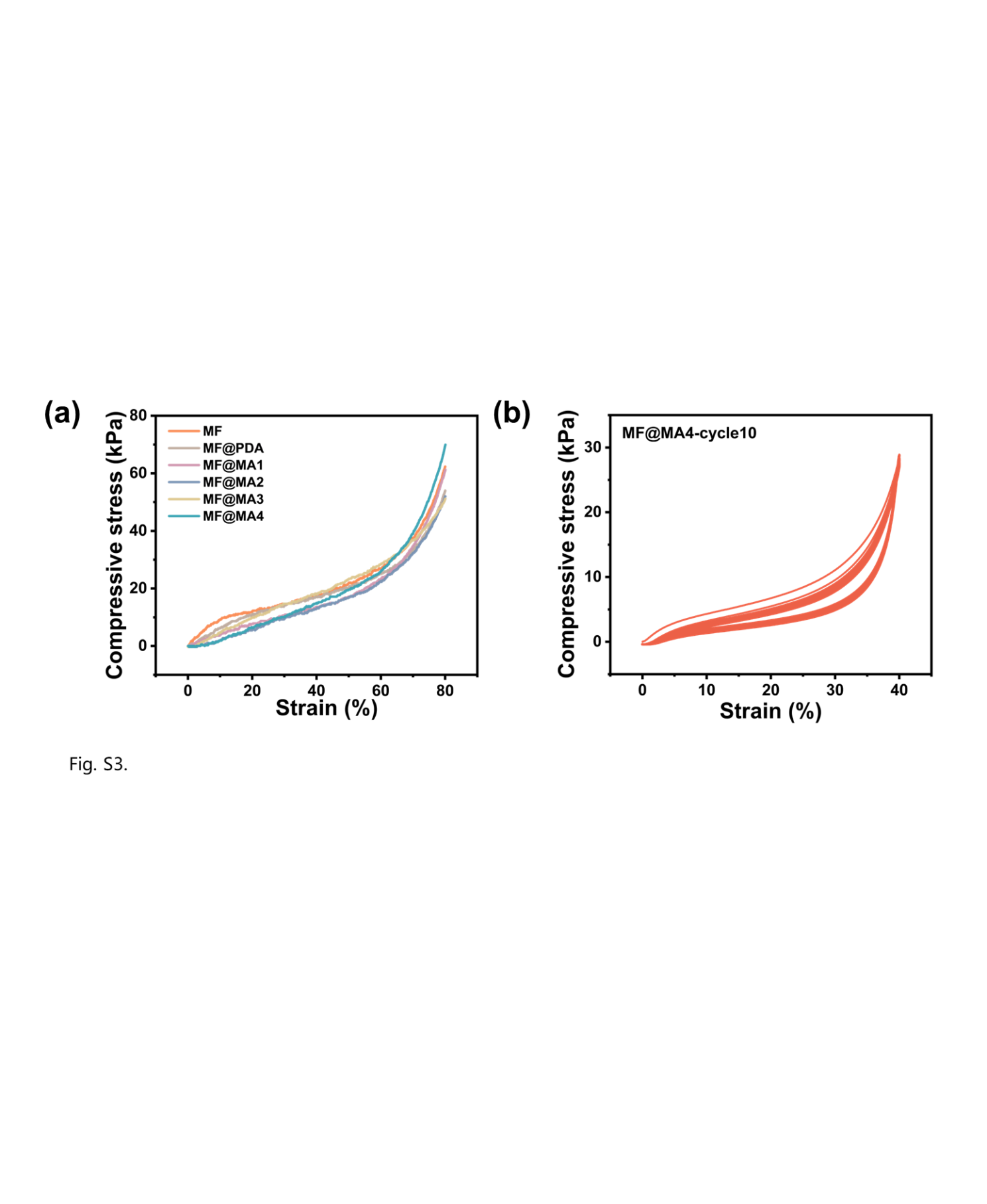


**Figure S3**. (a) Compressive stress-strain curves of MF@MA. (b) The compressive cycle curves of the MF@MA4 at 40% strain.


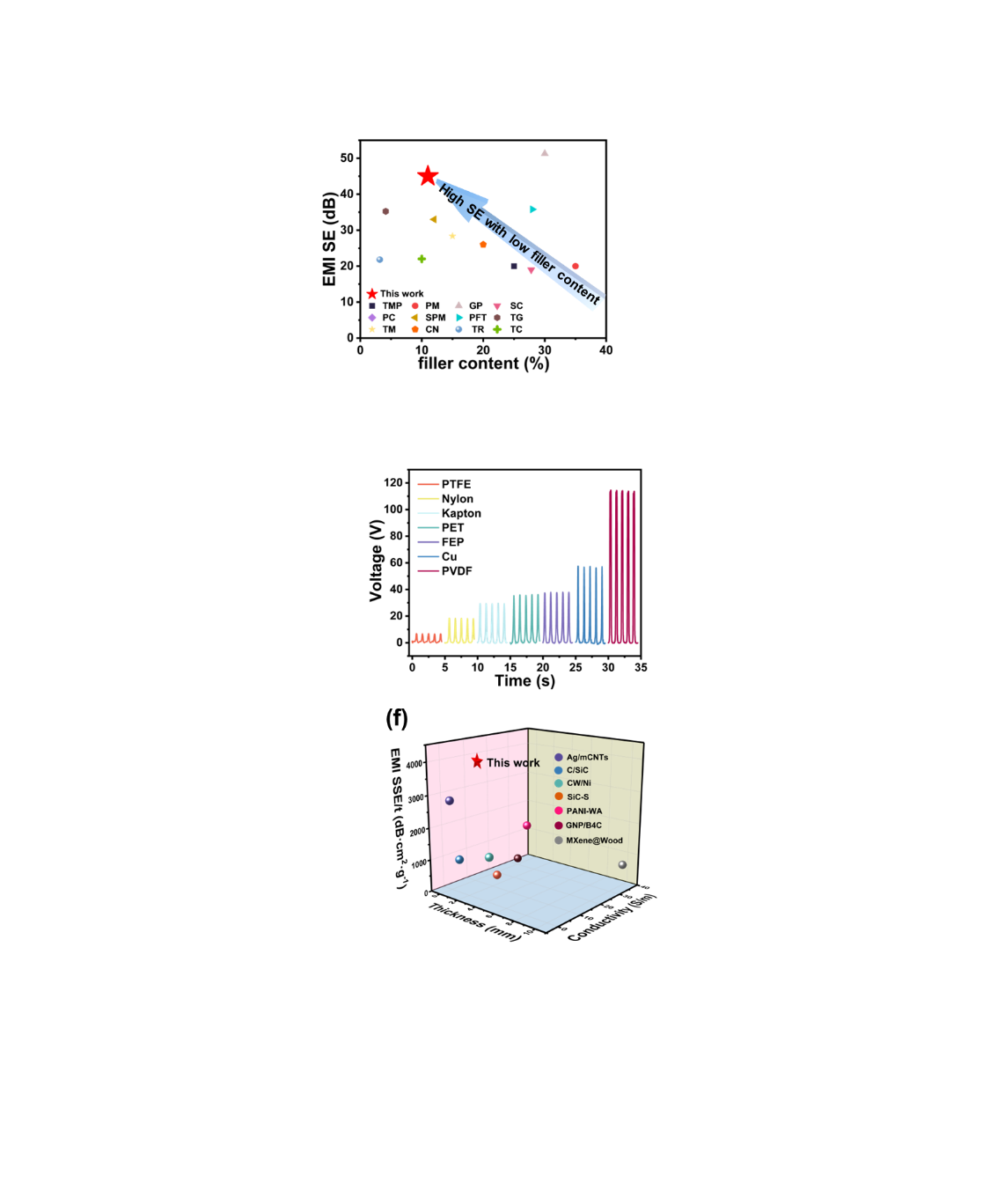


**Figure S4.** A comparison of EMI shielding performances between MF@MA and other reported materials.

**
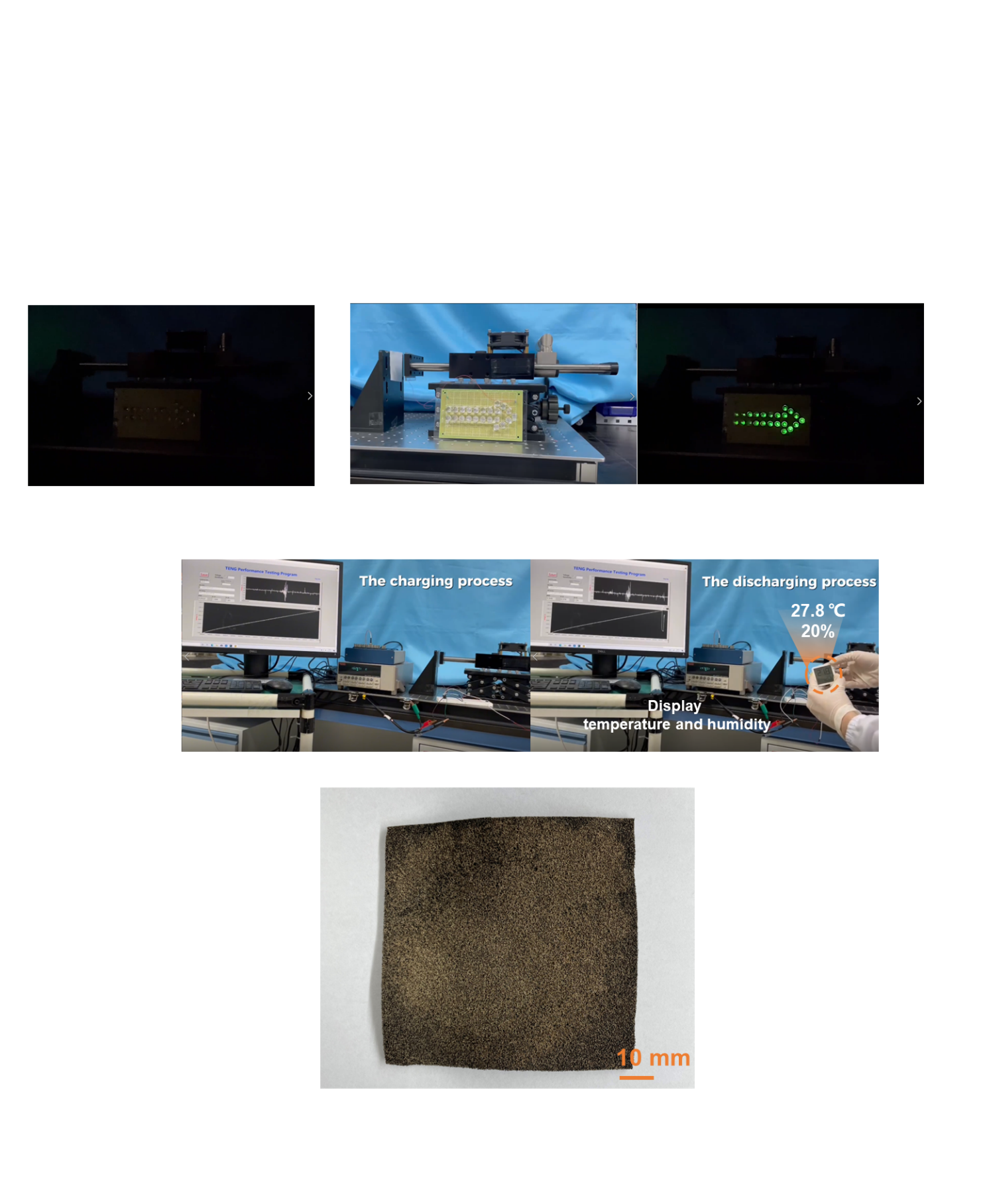
**

**Figure S5.** Optical diagram of MF@MA4 with a size of 100 mm ×100 mm.


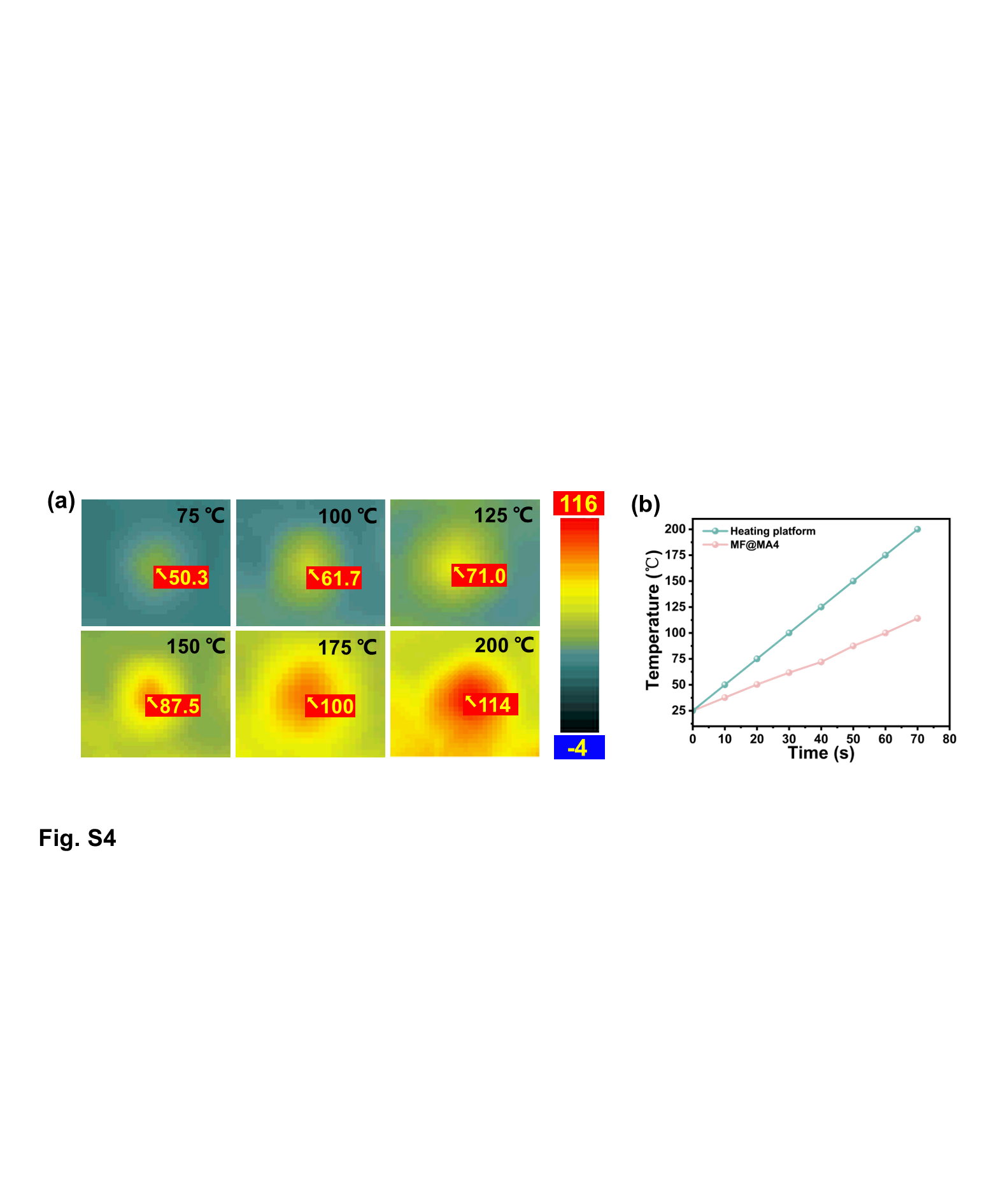


**Figure S6.** (a) Infrared images and (b) corresponding curves of MF@MA4 on the heating platform at different temperatures.

**
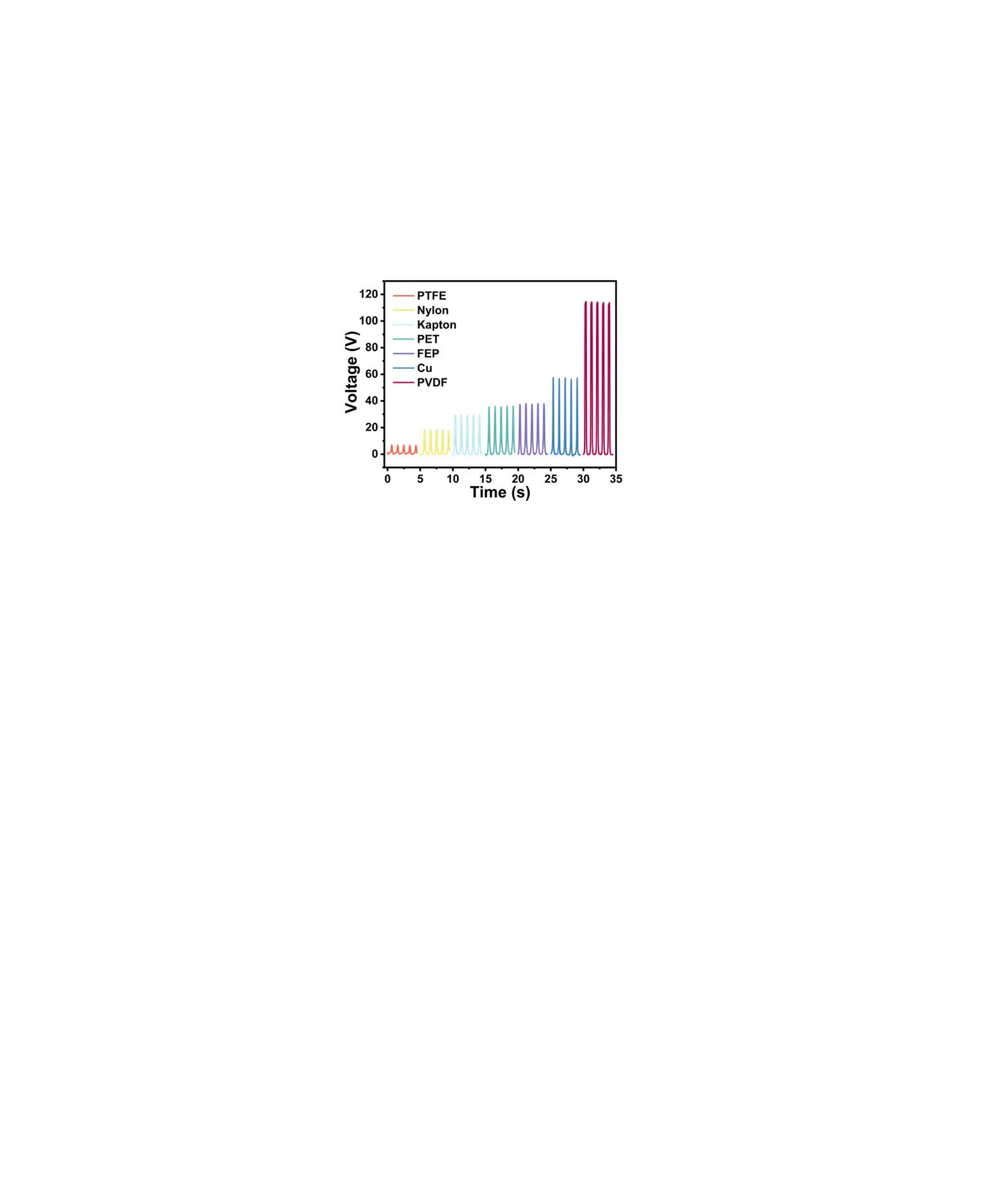
**

**Figure S7.** Output voltages of the MF@MA-TENG cooperated with PTFE, Nylon, Kapton, PET, FEP, Cu, and PVDF.


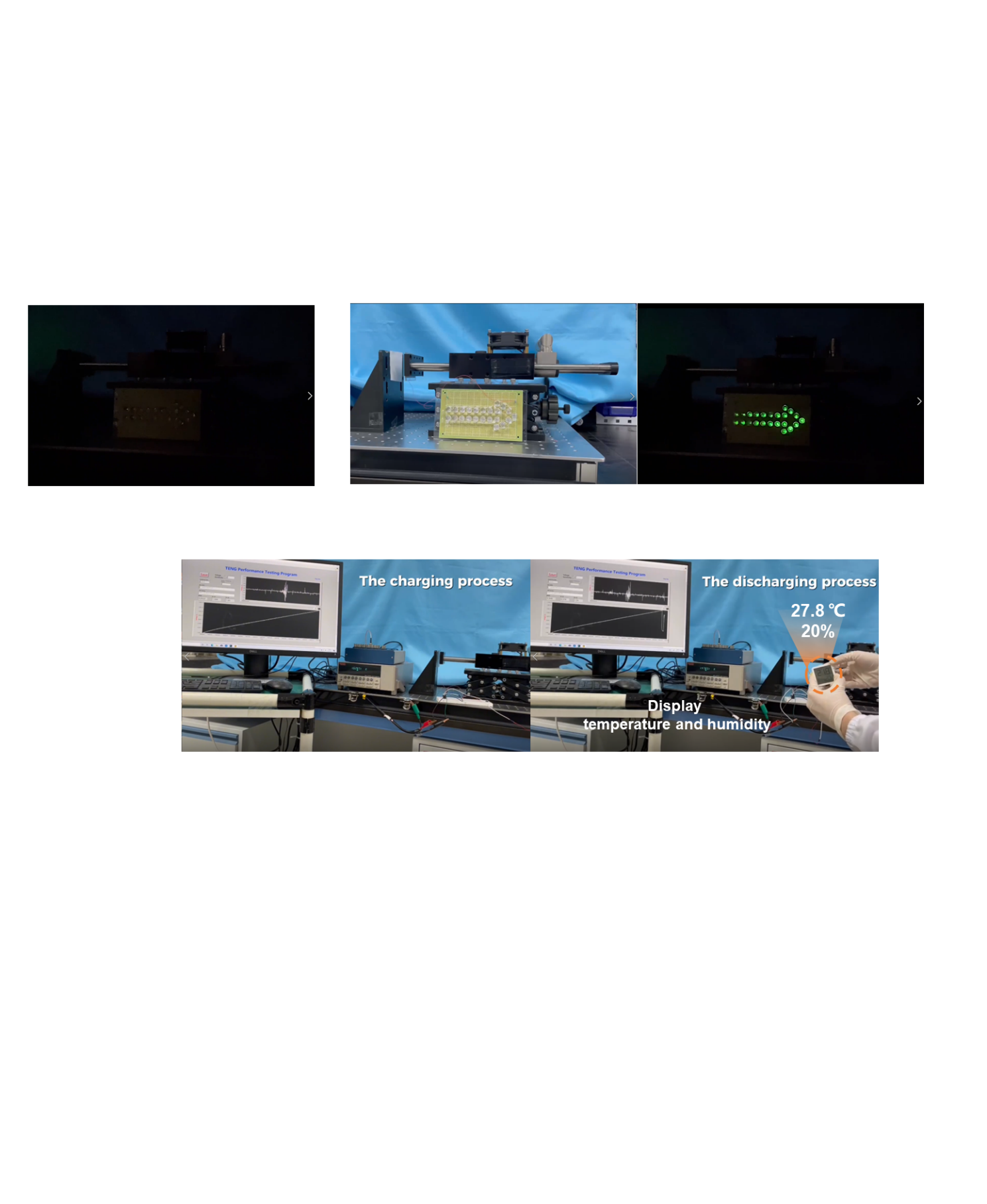


**Figure S8.** The photograph of driving all indicator lights by the MF@MA-TENG.


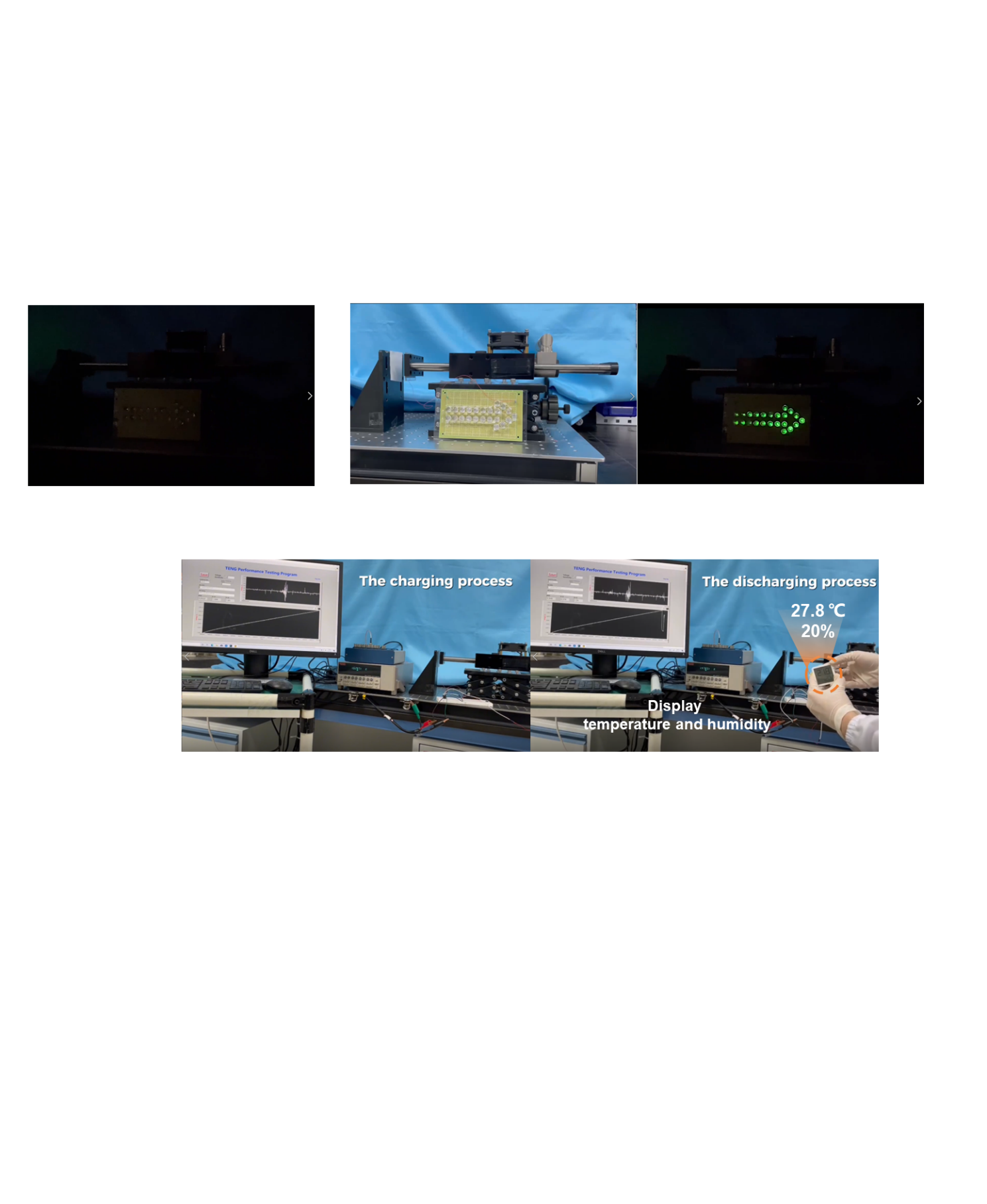


**Figure S9.** Digital image of driving a thermohygrometer by the MF@MA-TENG.

**Table S1.** The basic parameters of the composite foams.

| Samples | Density (g/cm^3^) | Electrical conductivity (S/m) |
| --- | --- | --- |
| MF@PDA | 0.022 | 0.003 |
| MF@MA1 | 0.034 | 0.012 |
| MF@MA2 | 0.045 | 0.153 |
| MF@MA3 | 0.053 | 4.000 |
| MF@MA4 | 0.060 | 4.444 |

**Table S2.** A comparison of EMI shielding performances between MF@MA and reported materials.

| Materials | SE_T_ (dB) | filler content (%) | Ref. |
| --- | --- | --- | --- |
| TPU/MMT/PPy | 20 | 25 | [1] |
| PU/MWCNT | 25 | 20 | [2] |
| GnP30@PDMS foam | 51.26 | 30 | [3] |
| SBS-g-MAH/CB | 19 | 27.8 | [4] |
| PVDF-CIP | 20 | 50 | [5] |
| S-PS/PVDF/Mx | 33 | 12 | [6] |
| PANI/FMWCNT/TPU | 35.8 | 28 | [7] |
| TPU/G | 35.23 | 4.14 | [8] |
| TPU/MPTU | 28.35 | 15 | [9] |
| CNT@PDMS/NW | 26 | 20 | [10] |
| TPU/RGO | 21.8 | 3.17 | [11] |
| TPU/CNT | 22 | 10 | [12] |
| MF@MA3 | 41.72 | 8.3 | **This work** |
| MF@MA4 | 48.32 | 11 |  |

**Table S3.** A comparison of SSE/t between MF@MA and other reported materials.

| Materials | Thickness (mm) | Conductivity (S/m) | SSE/t (dB·cm^2^·g^-1^) | Ref. |
| --- | --- | --- | --- | --- |
| SiC-S | 2.55 | 10 | 421.36 | [13] |
| GNP/B4C | 1.5 | 24 | 503.21 | [14] |
| MXene@Wood | 10 | 37 | 666.7 | [15] |
| C/SiC  CW/Ni | 0.41  3.5 | 2  3 | 948.18  1258.7 | [16]  [17] |
| PANI-WA | 3 | 22.07 | 1771.2 | [18] |
| Ag/mCNTs | 0 | 0.12 | 2811.78 | [19] |
| WCF/MXene | 2.68 | 1.72 | 4299.17 | [20] |
| GO nanosheets | 0.003 | 0.001 | 5000 | [21] |
| PP/MWCNT | 1.654 | 9.31 | 5421 | [22] |
| RGO/PI | 0.6 | 35 | 5678 | [23] |
| PMC_3_C | 0.168 | 9.08 | 6710 | [24] |
| MF@MA3 | 2 | 4 | 3935.84 | **This work** |
| MF@MA4 | 2 | 4.44 | 4026.67 |  |

**Table S4.** A comparison of power density between MF@MA-TENG and other reported TENGs.

| Materials | Power density (mW/m^2^) | Ref. |
| --- | --- | --- |
| Y-TENG | 0.17 | [25] |
| R-TENG | 11.5 | [26] |
| TIS-TENG | 32.5 | [27] |
| LMA-Ecoflex TENG | 130 | [28] |
| F-DLC TENG | 69.5 | [29] |
| GO-CNT HA-TENG | 288 | [30] |
| MXene CMC aerogel-based TENG | 402.94 | [31] |
| sw-TPU based TENG | 500 | [32] |
| MF@MA-TENG | 514.28 | **This work** |

**References**

[1] A.M. dos Santos, C. Merlini, S.D.A.S. Ramôa, G.M.O. Barra, Polym. Compos. **2020**, 41, 2003.

[2] N. Joseph, S.K. Singh, R.K. Sirugudu, V.R.K. Murthy, S. Ananthakumar, M.T. Sebastian, Mater. Res. Bull. **2013**, 48, 1681.

[3] S. Anand, M.C. Vu, D. Mani, J.-B. Kim, T.-H. Jeong, M.A. Islam, S.-R. Kim, Chem. Eng. J. **2023**, 462, 142017.

[4] L. Wu, D. Yao, X. Gao, Z. Yu, X. Wang, Y. He, Y. Zhu, C. Lu, K. Li, Smart Mater. Struct. **2020**, 29, 125018.

[5] N. Joseph, M. Thomas Sebastian, Mater. Lett. **2013**, 90, 64.

[6] J. Wang, K. Yang, H. Wang, H. Li, Eur. Polym. J. **2021**, 151, 110450.

[7] A.P. Sobha, P.S. Sreekala, S.K. **2017**, 113, 168.

[8] K. Ba, M. Zhang, X. Wang, P. Xu, W. Song, C. Wang, W. Yang, Y. Liu, Diamond Relat. Mater. **2023**, 131, 109585.

[9] M. Lin, J. Lin, L. Bao, Compos. Pt. A-Appl. Sci. Manuf. **2020**, 138, 106022.

[10] Z. Zhou, Z. Wang, X. Han, J. Pu, [Holzforschung](https://www.x-mol.com/paper/journal/74701?r_detail=1523695580710543360) **2022**, 76, 299.

[11] Q. Jiang, X. Liao, J. Li, J. Chen, G. Wang, J. Yi, Q. Yang, G. Li, Compos. Pt. A-Appl. Sci. Manuf. **2019**, 123, 310.

[12] S. Ramôa, G. Barra, R. Oliveira, M. Oliveira, M. Cossa, B. Soares, Polym. Int. **2013**, 62, 1477.

[13] S. Li, D. Liu, W. Li, G. Sui, ACS Sustain. Chem. Eng. **2020**, 8, 435.

[14] Y. Q. Tan, H. Luo, X. S. Zhou, S. M. Peng, H. B. Zhang, RSC Adv. 2**018**, 8, 39314.

[15] M. Zhu, X. Yan, H. Xu, Y. Xu, L. Kong, Carbon **2021**, 182, 806.

[16] Z. Zhang, L. Cheng, J. Tan, W. Yang, Ceram. Int. **2021**, *47*, 23942.

[17] Z. Dai, C. Hu, Y. Li, Z. Wang, Y. Wei, W. Zhang, J. Xu, S. Fu, X. Lin, ACS Appl. Nano Mater. **2023**, 6, 13646.

[18] J. Chen, Z. Zhu, H. Zhang, S. Tian, S. Fu, Mater. Des. **2021**, 204**,** 109695.

[19] L. Hu, Z. Kang, Appl. Surf. Sci. **2021**, 568, 150845.

[20] J. Zhai, C. Cui, A. Li, R. Guo, C. Cheng, E. Ren, H. Xiao, M. Zhou, J. Zhang, Ceram. Int. **2022**, 48, 13464,.

[21] M. Goodarzi, G. Pircheraghi, New J. Chem. **2024**, 48, 3539.

[22] Z.-I. Lin, C.-W. Lou, Y.-J. Pan, C.-T. Hsieh, C.-H. Huang, C.-L. Huang, Y.-S. Chen, J.-H. Lin, Compos. Sci. Technol. **2017**, 141, 74.

[23] G. K. Sharma, N. R. James, Synth. Met. **2023**, 296, 117376,.

[24] C. Xie, Y. Wang, W. Wang, D. Yu, Colloid Surf. A-Physicochem. Eng. Asp. **2022**, 651, 129713.

[25] F. Xing, Z. Ou, X. Gao, B. Chen, Z.L. Wang, Adv. Funct. Mater. **2022**, 32, 2205275.

[26] Q. Fu, Y. Liu, T. Liu, J. Mo, W. Zhang, S. Zhang, B. Luo, J. Wang, Y. Qin, S. Wang, S. Nie, Nano Energy **2022**, 102, 107739.

[27] Y. Xie, T. Shan, R. Chen, M. Zhang, S. Sun, X. Jian, H.-Y. Mi, C. Liu, C. Shen, Nano Energy **2023**, 116, 108786.

[28] S. Nayak, Y. Li, W. Tay, E. Zamburg, D. Singh, C. Lee, S.J.A. Koh, P. Chia, A.V.-Y. Thean, Nano Energy **2019**, 64, 103912.

[29] J. Zhou, M. Gao, J. Choi, ACS Appl. Electron. Mater. **2023**, 5, 2853.

[30] T. Huang, Y. Long, Z. Dong, Q. Hua, J. Niu, X. Dai, J. Wang, J. Xiao, J. Zhai, W. Hu, Adv. Sci. **2022**, 9, 2204519.

[31] F. Hu, J. Zeng, J. Li, B. Wang, Z. Cheng, T. Wang, K. Chen, ACS Appl. Mater. Interfaces **2022**, 14, 14640.

[32] J. Li, M. Zhang, G. Ni, H.Y. Mi, B. Dong, C. Liu, C. Shen, J. Appl. Polym. Sci. **2022**, 140, 53351.
